# Lower Extremity Salvage Through Dextranomer Debridement and Evolution Toward a Functional Pincer in a Diabetic Foot: Experience in Rural Surgery

**DOI:** 10.7759/cureus.116088

**Published:** 2026-09-11

**Authors:** Kassandra Fabiola Hernandez Garcia

**Affiliations:** 1 General Surgery, Hospital Universitario, Universidad Autónoma de Nuevo León (UANL), Monterrey, MEX

**Keywords:** autolytic debridement, dextranomer, diabetic foot, foot infection, granulation tissue, limb salvage, rural surgery, wound debridement

## Abstract

Necrotizing infections of the diabetic foot associated with peripheral vasculopathy remain a critical challenge in emergency and general surgical practice, particularly in low-resource, second-level rural hospitals that lack access to reconstructive subspecialties. We report the case of a 57-year-old man with type 2 diabetes mellitus and peripheral vascular disease who presented to a rural, second-level public hospital in Linares, Nuevo León, Mexico, with a deep, contaminated surgical wound following a prior amputation of the second and third toes of the left foot performed at another rural facility. On admission, he had poor glycemic control, local pain, and a wound bed with abundant slough, debris, fibrin, and liquefactive necrosis threatening progression toward the lateral metatarsals. Management consisted of titrated insulin therapy, broad-spectrum systemic antimicrobials (imipenem and vancomycin), and formal surgical debridement with mechanical washout using saline and iodine solution. Postoperatively, topical dextranomer microbeads (Debrisan) were applied serially to the wound bed to promote continuous autolytic debridement. Dextranomer controlled exudate and mechanically removed fibrin by capillary action without impairing angiogenesis, halting necrotic progression and accelerating the transition to a clean, flat wound bed with highly viable granulation tissue. Guided healing and biological retraction ultimately produced a functional interdigital pincer deformity that preserved plantar weight-bearing, avoiding a major proximal amputation after an 18-day hospital stay. This case illustrates that dextranomer is a low-cost, high-utility adjunct to aggressive surgical debridement for complex, infected diabetic foot wounds in resource-limited rural settings, and that a functional pincer-type anatomical outcome can preserve independent ambulation while avoiding infracondylar amputation.

## Introduction

Necrotizing infections of the diabetic foot associated with peripheral vasculopathy represent a critical challenge in emergency departments, particularly in peripheral second-level units that operate with limited resources and lack access to reconstructive subspecialties [1,2]. When postsurgical infectious processes carry a high risk of progression to limb loss, the general surgeon must establish aggressive therapeutic strategies aimed at tissue damage control [3]. In this scenario, timely debridement combined with advanced adjunctive therapies not only limits the systemic inflammatory response and deep bacterial invasion but also promotes a biological wound bed suitable for directed healing, avoiding major infracondylar amputations and preserving the residual functional length of the extremity [4,5].

Dextranomer, a hydrophilic dextran polymer supplied as microbeads (Debrisan), has been used since the late 1970s as a molecular adjunct for cleansing contaminated and infected wounds; its microgranules absorb exudate, bacteria, and necrotic debris by capillary action, promoting autolytic debridement while sparing angiogenesis and healthy granulation tissue [6,7]. We describe the use of dextranomer as an adjunct to surgical debridement in a patient with a complex, infected diabetic foot wound managed in a rural, second-level hospital, with evolution toward a functional pincer-type foot that preserved ambulation [8]. 

Unlike passive dressings that primarily manage exudate, dextranomer actively drives autolytic debridement between surgical sessions without disturbing granulation tissue or neoangiogenesis, a property particularly valuable in rural settings lacking access to daily surgical debridement or advanced technologies such as negative-pressure wound therapy or bioengineered skin substitutes.

For this report, a functional pincer is defined as a residual forefoot configuration resulting from guided secondary-intention healing after partial digital amputation, in which the remaining medial and lateral forefoot structures retract and approximate one another, recreating an opposable, claw-like contact surface that preserves plantar weight-bearing and allows independent ambulation without a more proximal amputation.

## Case presentation

We present the case of a 57-year-old man with a history of type 2 diabetes mellitus and peripheral vascular disease who was evaluated at a rural, second-level unit of the Ministry of Health in Linares, Nuevo León, Mexico. The patient had previously undergone amputation of the second and third toes of the left foot at another rural facility. Because of a lack of clinical improvement and progressive local deterioration, he sought specialized management at the Department of General Surgery of the reporting institution.

On admission, the patient had poor glycemic control (capillary glucose 306 mg/dL, HbA1c 10%) and leukocytosis (26,200 cells per microliter), with local pain and a complicated surgical wound. The wound bed measured approximately 5 to 7 cm along its longitudinal axis, extending from the first interdigital commissure toward the plantar and posterior metatarsal region, and 2 to 4 cm along its transverse axis, with a cavernous tract extending toward the bone and deep soft tissue and secondary necrotic and ischemic extension of approximately 3 to 4 cm at the base of the second and third toes.

The ankle-brachial index was 0.6, consistent with peripheral vascular disease; Doppler ultrasound and angiography were not available at this rural facility. C-reactive protein testing and wound cultures could not be obtained due to a lack of the necessary reagents and culture capability at the time of admission. A comprehensive treatment protocol was implemented, consisting of metabolic stabilization with titrated insulin doses, broad-spectrum systemic antimicrobial support (imipenem and vancomycin) [9], and formal surgical management in the operating room.

Surgical debridement was performed on hospital day 1, including thorough wound exploration, extensive mechanical washout with saline and iodine solution, and radical debridement of denatured tissue, fibrin, and necrotic foci (Figure 1).

**Figure 1:**
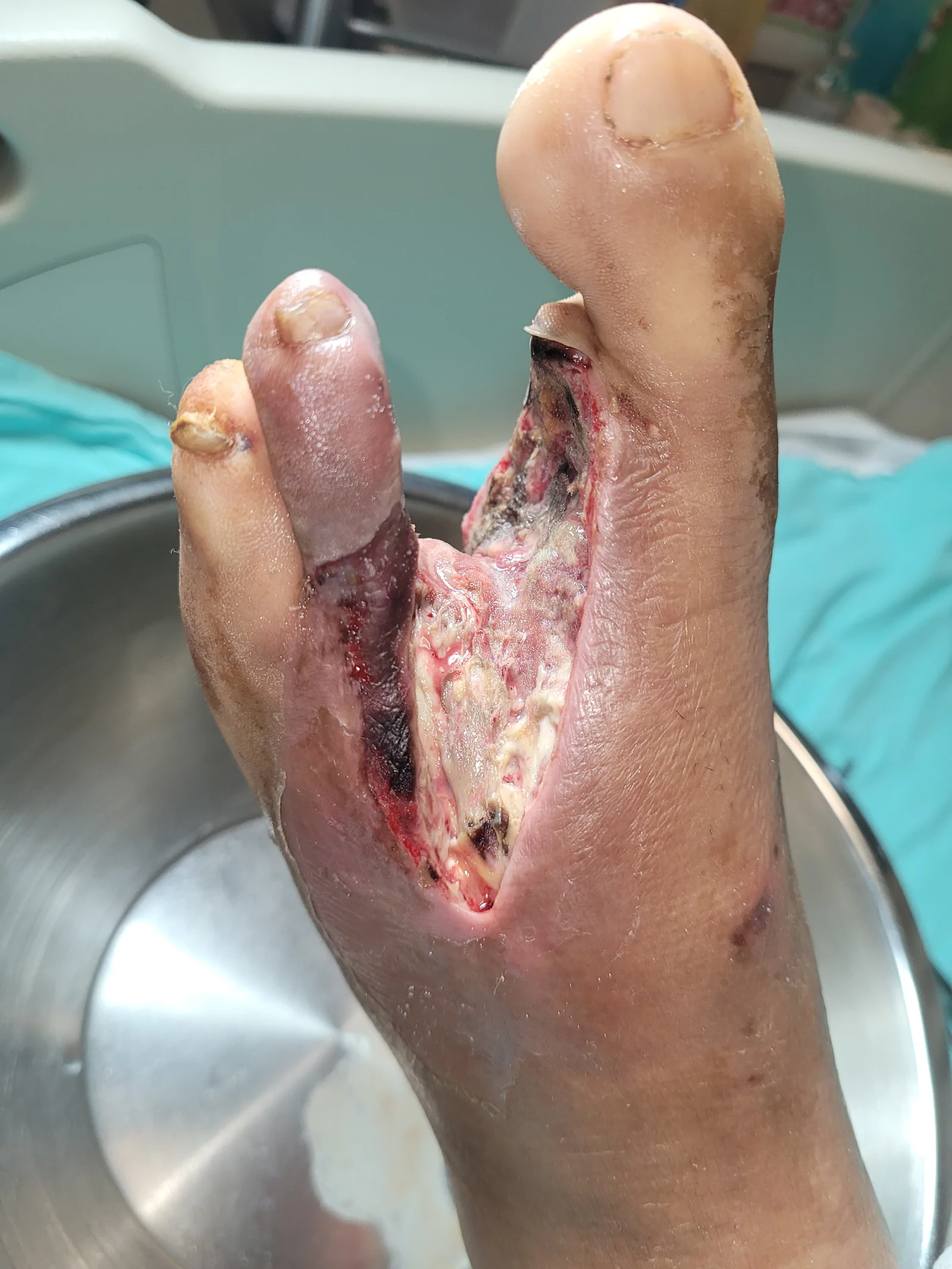
Clinical photograph from the first week of hospitalization, showing the wound bed after radical surgical debridement, with abundant slough, fibrin, and necrotic tissue.

Topical dextranomer microbeads (Debrisan) were obtained and started on day 2, applied serially to the wound bed to promote continuous autolytic debridement; dextranomer controlled exudate and removed fibrin by capillary action without impairing angiogenesis. Due to persistent necrotic progression, a second surgical intervention was performed on day 7, requiring amputation of the fourth toe along with more extensive debridement; from that point onward, management continued with bedside wound irrigation and serial dextranomer application. Viable granulation tissue was first documented on day 11, and by the second week of treatment, the wound bed had transitioned to a completely flat, clean surface with highly viable granulation tissue (Figure 2).

**Figure 2:**
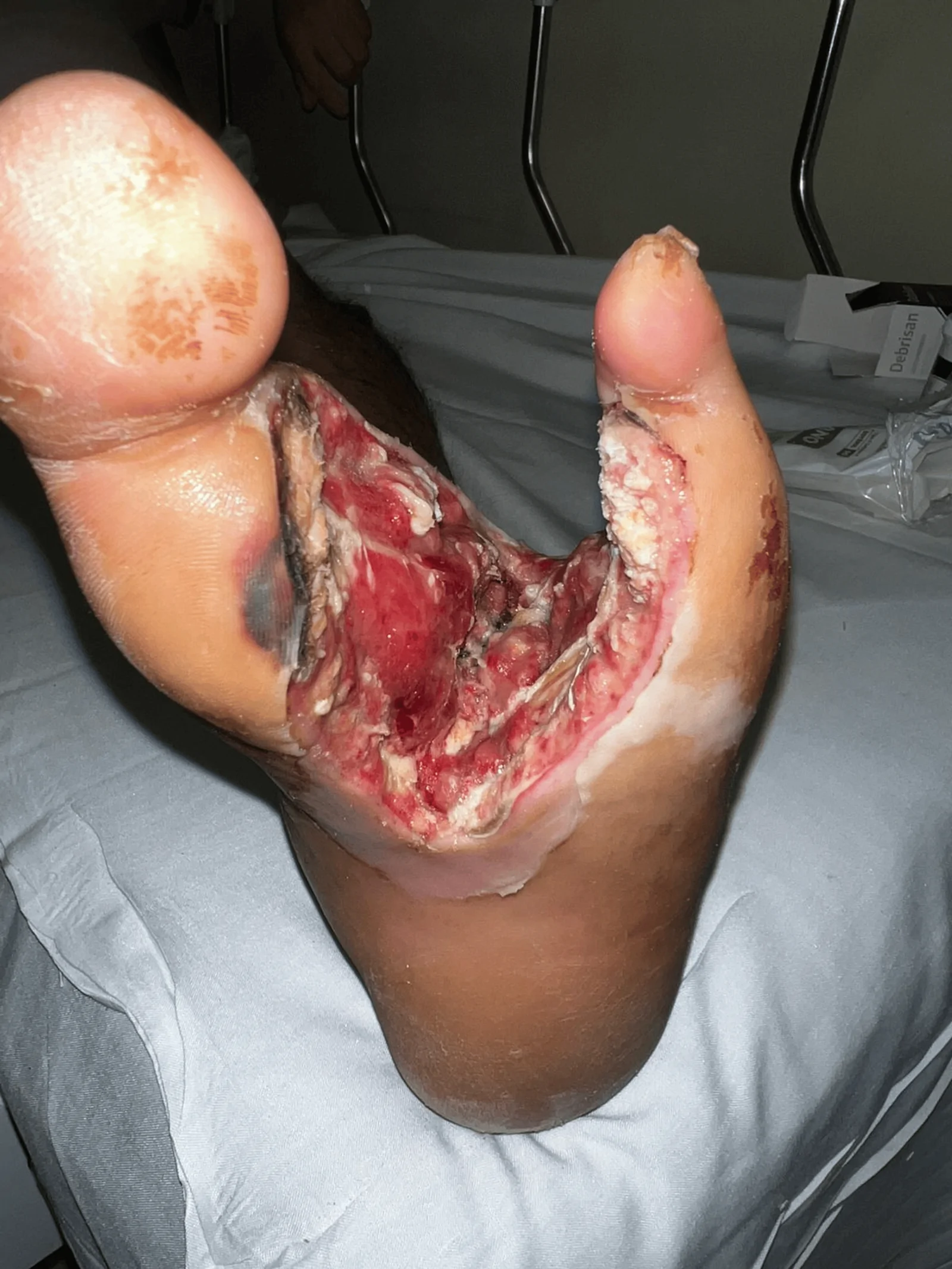
Clinical photograph from the second week of hospitalization, showing transition to a flat, clean wound bed with highly viable granulation tissue after serial dextranomer application.

Wound evolution and anatomical configuration were monitored throughout the hospital stay. Guided healing and biological wound retraction ultimately consolidated into an anatomical remodeling that resulted in a functional pincer configuration, preserving plantar support. The patient was discharged on hospital day 18, at which point capillary glucose was 104 mg/dL and the leukocyte count had decreased to 10,300 cells per microliter, without requiring a major proximal amputation. At discharge, the wound bed showed clean, well-demarcated margins with active granulation tissue and a decreasing wound area, approximately 6 to 8 cm longitudinally by 2.5 to 4 cm transversely, with a residual depth of 1 to 2 cm at the proximal apex, plus a smaller secondary bed of 3 to 4 cm and a focal 0.8 to 1 cm granulating point lesion at the hallux base. At one-month follow-up after discharge, the patient returned with a healed, stable, functional pincer configuration, with preserved plantar weight-bearing and independent ambulation (Figure 3).

**Figure 3:**
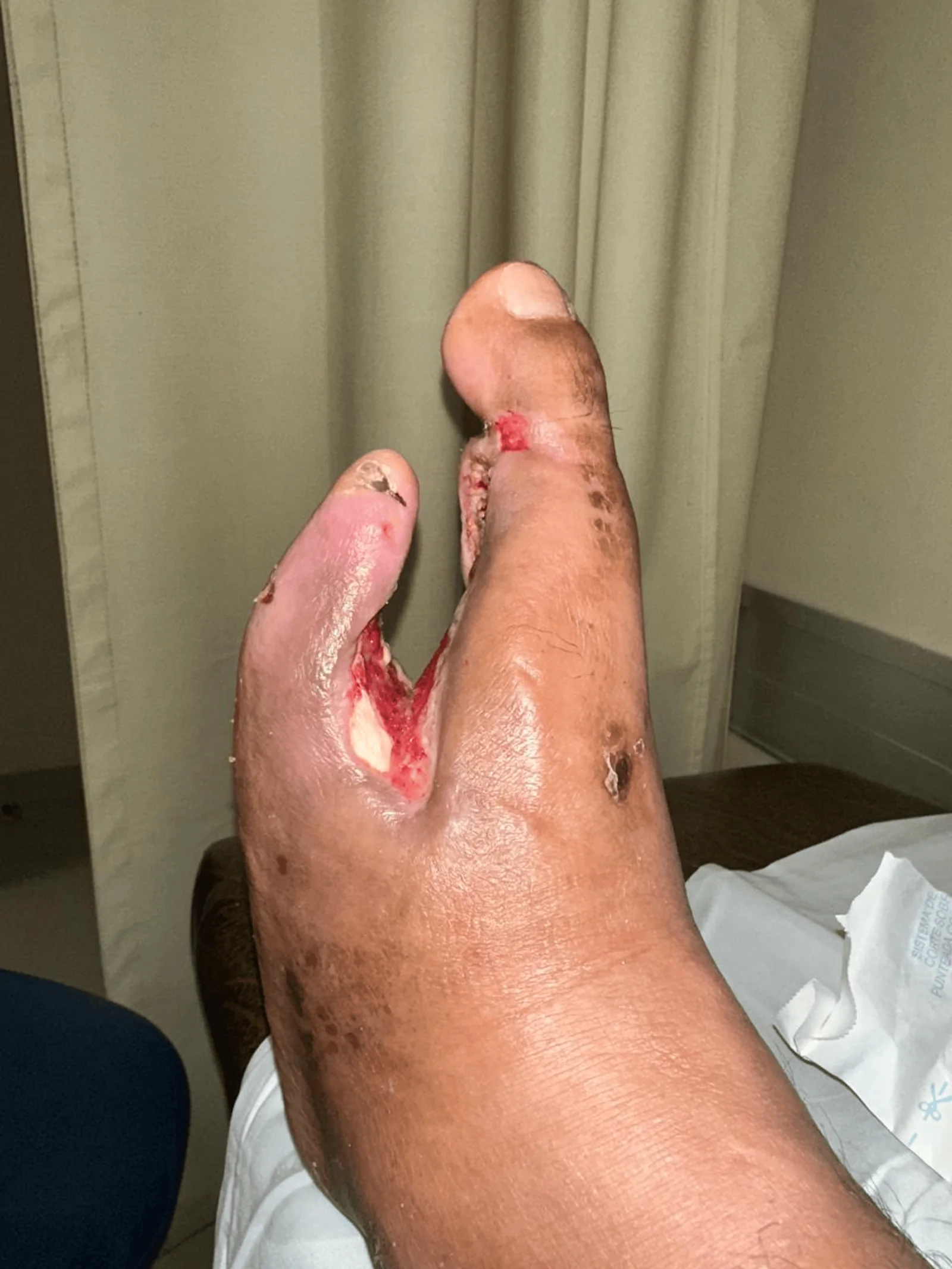
Clinical photograph obtained one month after hospital discharge, showing the healed, functional interdigital pincer configuration with preserved plantar support.

## Discussion

Necrotizing infections of the diabetic foot in patients with peripheral vasculopathy pose a particular challenge for general surgeons practicing in rural, resource-limited settings, where access to plastic and reconstructive surgery, hyperbaric oxygen therapy, and advanced wound care technologies is frequently unavailable [2]. In this context, aggressive surgical debridement remains the cornerstone of source control. Still, the choice of adjunctive wound care can materially influence whether a patient progresses toward limb salvage or major amputation [10].

Dextranomer is a highly hydrophilic dextran polymer manufactured as porous microbeads that, once in contact with exudate, swell and draw bacteria, fibrin, and necrotic debris into the interbead spaces by capillary action, thereby achieving mechanical and autolytic debridement while leaving healthy granulation tissue and neoangiogenesis undisturbed [6,7]. Early clinical evaluations demonstrated that dextranomer effectively cleanses infected and malodorous wounds and accelerates the appearance of healthy granulation tissue [11,12], findings that are consistent with the clinical course observed in this patient, in whom serial dextranomer application controlled exudate, cleared fibrin, and promoted a rapid transition to a viable, granulating wound bed.

From a resource-utilization standpoint, dextranomer is an inexpensive, easy-to-apply adjunct that does not require specialized equipment or subspecialty training, making it particularly well suited to second-level rural hospitals where negative-pressure wound therapy, bioengineered skin substitutes, or microsurgical reconstruction are not readily available. In this case, the combination of radical surgical debridement, systemic antimicrobial therapy, tight glycemic control, and serial dextranomer application prevented progression to an infracondylar amputation and instead allowed spontaneous anatomical remodeling into a functional interdigital pincer foot that preserved plantar weight-bearing and independent ambulation [8].

This report is limited by its retrospective, single-case design, which precludes broader conclusions about the generalizability of dextranomer therapy in diabetic foot infections. Additionally, resource constraints at this rural facility precluded C-reactive protein testing, wound culture, and vascular imaging with Doppler ultrasound or angiography beyond ankle-brachial index measurement, which limits the granularity of the infectious and vascular characterization presented. Nonetheless, it adds to a small but consistent body of literature supporting dextranomer as a valuable adjunct for the general surgeon managing complex, contaminated diabetic foot wounds outside of tertiary reconstructive centers.

## Conclusions

Dextranomer (Debrisan) is a highly useful and cost-effective molecular tool for managing complex, contaminated diabetic foot wounds in the rural setting. When combined with aggressive surgical debridement and advanced wound care protocols, it can effectively prevent progression to disarticulation or major infracondylar amputation.

In this patient, the consolidation of a functional pincer-type anatomical sequela preserved independent ambulation, demonstrating that biological limb salvage is an achievable and priority goal for the general surgeon practicing at the second level of care, even in settings with limited access to reconstructive subspecialties.
